# On Railway and Other Injuries of the Nervous System

**Published:** 1867-08

**Authors:** 


On Railway and other Injuries of the Nervous System. By JOHN ERIC ERICHSEN, Fellow of the Royal College of Surgeons; Professor of Surgery and of Clinical Surgery in University College; Surgeon to University College Hospital; etc., etc., etc. Philadelphia: HENRY C. LEA. 1867.

This is a neatly published monograph of 103 octavo pages. It comprises six lectures delivered to the students attending the University College Hospital in the spring of 1866. The first lecture is mainly introductory, and relates to the importance of injuries of the spine and the different views concerning them. The second treats of the effects of severe blows on the spine. The third, of concussion of the spine from slight injuries. The fourth, of concussion of the spine from general shock; and of twists and wrenches. The fifth, of the symptoms and pathology of concussion of the spine. The sixth, of the diagnosis, prognosis, and treatment of injuries of the spine.

Throughout the work, the lecturer has reference to such injuries and concussions as result from railroad accidents and casualties. The frequency of such accidents renders the present work timely and important, to all classes of medical men.

For sale by W. B. KEEN & Co., 148 Lake St., Chicago.

